# Characterization of Mixing Performance Induced by Double Curved Passive Mixing Structures in Microfluidic Channels

**DOI:** 10.3390/mi12050556

**Published:** 2021-05-13

**Authors:** Ingrid H. Oevreeide, Andreas Zoellner, Bjørn T. Stokke

**Affiliations:** 1 Division of Biophysics and Medical Technology, Department of Physics, NTNU The Norwegian University of Science and Technology, NO-7491 Trondheim, Norway; ingrid.h.ovreeide@ntnu.no; 2 Independent Researcher, Palo Alto, CA 94301, USA; amz@alumni.stanford.edu

**Keywords:** passive mixing, curved mixing structure, confocal microscopy, mixing efficiency

## Abstract

Functionalized sensor surfaces combined with microfluidic channels are becoming increasingly important in realizing efficient biosensing devices applicable to small sample volumes. Relaxing the limitations imposed by laminar flow of the microfluidic channels by passive mixing structures to enhance analyte mass transfer to the sensing area will further improve the performance of these devices. In this paper, we characterize the flow performance in a group of microfluidic flow channels with novel double curved passive mixing structures (DCMS) fabricated in the ceiling. The experimental strategy includes confocal imaging to monitor the stationary flow patterns downstream from the inlet where a fluorophore is included in one of the inlets in a Y-channel microfluidic device. Analyses of the fluorescence pattern projected both along the channel and transverse to the flow direction monitored details in the developing homogenization. The mixing index (MI) as a function of the channel length was found to be well accounted for by a double-exponential equilibration process, where the different parameters of the DCMS were found to affect the extent and length of the initial mixing component. The range of MI for a 1 cm channel length for the DCMS was 0.75–0.98, which is a range of MI comparable to micromixers with herringbone structures. Overall, this indicates that the DCMS is a high performing passive micromixer, but the sensitivity to geometric parameter values calls for the selection of certain values for the most efficient mixing.

## 1. Introduction

Integration of microfluidics and microfluidic channels within several scientific domains have increased over the past few decades. This merger has led to the development of devices such as Lab-on-Chip, Organ-on-Chip, and sensors for the determination of various biological, chemical, and medical analytes, as well as applications as reactors [1,2,3,4]. Substantial advantages associated with working in the miniaturized regime, including microfluidics, motivates such developments. An example of favorable features includes the use of a reduced sample volume and read out time and an improved limit-of-detection (LOD). Although microfluidics come with several advantages, there are also challenges with operating in the micro-regime. One of the major disadvantages is the laminar flow regime encountered for fluid flow at the microscale. This reduces the impact of inertial forces over the viscous forces and yields low Reynold’s numbers. Typically, with a laminar flow, the only mixing that occur is by diffusion, which is a relatively inefficient mixing process. This limitation of laminar flows has led to the development of different mixing techniques to enhance the rate of mixing. The various fluid mixing strategies are grouped into processes referred to as either active or passive, where the active mixers require external energy input.

Active mixers use peripheral devices to drive the mechanism of the mixing process, e.g., based on acoustics, electrostatics, or other principles [5,6,7,8]. The peripherals thus deliver energy to the devices that is dissipated, which may lead to heating. Although the resultant mixing efficiencies are often over 90% for active mixers [9,10], the additional equipment increases the complexity of the system, cost, size and can in cases be harmful to biological samples due to the energy dissipation. These drawbacks have led to an increased focus on the development of passive micromixers and their optimization.

Passive mixers do not require energy supplied by peripherals, while at the same time they show a substantial increased mixing efficiency compared to diffusion driven process. Several different strategies have been implemented ranging from changing the 2D geometry of the channels [11,12,13,14,15], including obstructions [16,17,18,19,20] or adding different structures to one or more of the channel surfaces [21,22,23,24,25,26,27,28,29,30], amongst others [31,32,33,34,35,36]. The slanted groove mixer (SGM) [37] exploiting fabricated structures in the ceiling of the microfluidic channels are among the designs that have attracted the most attention.

The SGM involves the addition of slanted bar structures localized at one of the walls of a microfluidic channel [37]. Stroock et al. (2002) expanded on this design by the addition of an asymmetric arm to the SGM, resulting in the well-known staggered herringbone mixer (SHM) [38]. In their original work, they reported that addition of an asymmetric element resulted in a more efficient passive mixer due to the development of mixing via chaotic advection, compared to channels containing no mixing elements or the SGM.

Following the initial publication of the SHM, several studies have been performed focusing on the variation of different geometrical parameters of the passive mixing structure to enhance their performance. Yang et al. (2005) found that the height ratio and asymmetry index of the grooves (length of long arm to short arm) were the key parameters in influencing the mixing performance of the SHM [35]. An asymmetry index of 2/3 has been shown to be optimal [38,39,40] and a deeper groove yielded increased mixing efficiencies [35,41,42,43]. Furthermore, the groove depth was also identified as an important parameter, where a wider groove increased the mixing efficiency induced by the structure [44].

Stroock et al. (2002) also predicted that the use of SHM would increase the rate of a diffusion limited reaction, through the stirring of the boundary layer [38], which was further investigated by other research groups [45,46]. An application of this would be surface-based sensors, such as biosensors. Biosensors often require a certain minimum concentration of analyte receptor binding for the analyte to be sensed by an underlying readout principle, e.g., changes in the mass or refractive index adjacent to or connected to the sensor [47,48,49]. Passive mixing structures in microfluidic channels have been used in conjunction with surface biosensors to enhance analyte concentration at the sensor [50], thus decreasing the LOD and readout time. Lynn et al. implemented an SHM in the ceiling of a microfluidic channel, which, combined with a surface plasmon resonance (SPR) sensor, showed an increase in sensitivity, where different parameters varied the efficiency of the sensor [51,52,53,54,55].

An increase in the rate of surface coverage of analyte as induced by a microfluidic channel with mixing structures will decrease the channel length needed to increase the probability of an analyte reaching a surface sensor. Comparing this to its efficiency in mixing an optimal channel for mass transport and mixing, e.g., for diluting samples, can be calculated. A shorter channel and increased surface coverage rate would also allow for a reduction in the sample volume required for the minimum concentration necessary to obtain a signal beyond the signal-to-noise ratio of certain types of biosensors within reasonable time frames.

We previously reported on a comparison of the mixing efficiency of a curved passive mixing structure (CMS) with that of herringbone structures (HBS) and found that the CMS represented a more robust group of design geometries in maintaining efficient mixing than the HBS [56]. Specifically, we reported a mixing index (MI) 1 cm downstream from the inlet in the range of 0.85–0.99 for various parameter values of the designs in the CMS group, compared to the HBS group that resulted in an MI range of 0.74–0.98. These finding motivated further studies of passive mixing structure geometries based on our initial CMS design by the addition of an asymmetric element. The resulting structure had two curved structures, thus also resembling the HBS designs with its two branches, and with the location of the mixing elements along the channel, reverting the asymmetry location every second structure. In the following section, we describe the experimental characterization of the mixing performance, the change in MI and mass transfer abilities, of the adapted double curved mixing structure (DCMS) fabricated in the ceiling of microfluidic channels.

## 2. Materials and Methods

### 2.1. Fabrication

Microfluidic mixing channels were fabricated using a two-layer photo-lithographic method following the procedure previously outlined [56]. In brief, the master for the soft lithography was fabricated by spin-coating a negative photoresist (MrDWL40, Micro Resist Technologies) on a silicon wafer, baked at 90 °C following a stepwise 5 °C temperature ramping, patterning of the flow channel by exposing to a laser at 405 nm (MLA150 Maskless Aligner, Heidelberg) and post-exposure baked (PEB) at 90 °C. The second resist layer was spun onto layer one after cooling, and the mixing design exposed using the same procedure. The final mold was obtained by developing the exposed structure (MrDev 600, Micro Resist Technology) and was silanized to allow for easier removal of the PDMS. The PDMS (Sylgard 184, Dow Corning) was added at a 1:10 curing agent to elastomer weight, degassed, poured on the mold and then baked at 65 °C for 3 h. The final channels were peeled from the master, cut, and outlet and inlets were punched (Ø1.0 mm, Miltex Biopsy Punch). To complete the device, a microscope slide and the PDMS channel were activated using oxygen plasma and bonded together.

### 2.2. Passive Mixer Designs

**Figure 1:**
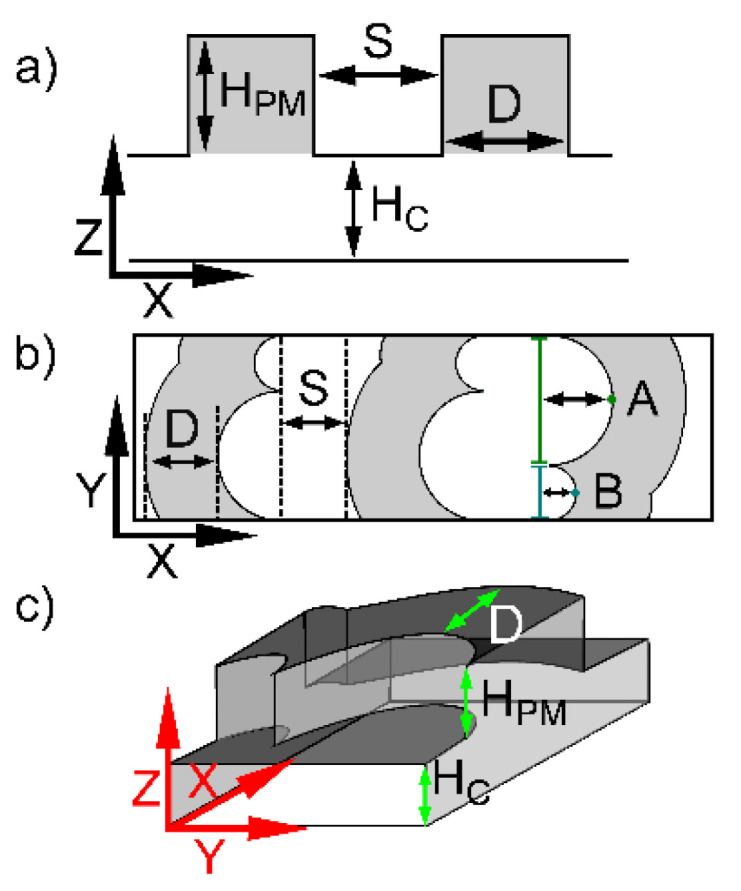
Schematic illustration of microfluidic channel designs showing relevant parameters. The overall transverse widths of each channel were 100 µm. (**a**) Side view of a channel design with passive mixing structures in the ceiling where the design parameters are as follows: H_c_ depicts the channel height; H_PM_ is the passive mixer height. (**b**) Top view depicting the passive mixer depth, D, and passive mixer spacing, S, for DCMS, where the short arm to perpendicular intersection is 30 µm (teal line) and long arm is 70 µm (green line). A = 35 µm and B = 15 µm. (**c**) 3D view of DCMS also depicting the location of H_c_, H_PM_ and D.

Two different groups of passive mixing designs were studied. These groups were a curved mixing structure (CMS) as introduced previously [56], and a double curved mixing structure (DCMS) extending from the CMS by adding a second curve to the overall geometry (Figure 1). A total of 16 different channels with passive mixing designs were fabricated for each group. The DCMS was divided into cycles of two structures, alternating the direction of the long and short curved structure for each cycle. Due to the varying groove pitch of the mixing structures, the number of structures within the overall channel length (1 cm) differed for each channel. The geometrical parameters are shown in Figure 1.

The various channels with the DCMS design were realized by selecting different channel heights (H_C_), and the passive mixing structure depth (D), spacing (S) and height (H_PM_) (Table 1).

In the following we refer to the parameters in the various mixing structure designs using the wafer they were produced on (W_1_–W_4_), the passive mixing design used (DCMS) followed by the PDV (1–4), e.g., W_1_ DCMS 1 would have the dimensions H_C_ = 20 µm, H_PM_ = 20 µm, and the Double Curved Mixing Structures (DCMS) would have D = 20 µm and S = 20 µm. Whilst W_4_ DCMS 4 would have the dimensions 40 µm, 60 µm, 120 µm and 240 µm, respectively.

### 2.3. Confocal Imaging

The flow pattern in the microfluidic channels with the various mixing structures were determined using confocal microscopy (Leica TCS SP5), employing fluorescein (Fluorescein sodium salt SigmaAldrich) included in the aqueous solutions injected to one of the Y-inlets as a reporter. The fluorescein solution and deionized water were added to two separate 5 mL syringes and were simultaneously injected (10 µL/min, Harvard apparatus syringe pump) via plastic tubes in the two separate inlets of the Y-channel. This resulted in a mixing flow rate of 20 µL/min and a Reynold’s number of ~4. XYZT-stack images were obtained for specific locations downstream from the inlet employing a 10× (NA = 0.4) HC PL Apo CS dry objective. The image acquisition process is further detailed in our previous study [56].

### 2.4. Data Processing

The acquired confocal XYZT-stacks along the microfluidic channel were analyzed using custom-designed MATLAB scripts. A virtual sensor volume was chosen between two mixing structures for each stack and the lowest and highest Z-level within the microfluidic channel was determined. This was also used as a basis for the analysis within the fluid layer adjacent to a most likely location of a transducing sensor element. After calculating the standard deviation of the fluorescence intensity at the inlet, the pixel intensities were normalized to 1, and each consecutive measurement for the same channel was then normalized to the inlet signal. We calculated the mixing index (MI) from the standard deviation of the normalized fluorescence intensity profile for each image volume along each channel using Equation (1):

(1) $$\mathrm{MI}=1-\frac{\sqrt{\frac{1}{N-1}\sum_{i=1}^{N}{\left(c_{i}-\bar{c}\right)}^{2}}}{{\sqrt{\frac{1}{N_{in}-1}\sum_{i=1}^{N_{in}}{\left(c_{i}-\bar{c}\right)}^{2}}}_{Inlet}}\;$$

In Equation (1), *N* is the number of pixels, *c_i_* is the pixel intensity, *c* is the mean pixel intensity, and *N_in_* is the number of pixels at the inlet. A completely unmixed channel has an MI of 0, whilst a completely mixed channel has a value of 1. The MI was empirically observed to equilibrate towards the completely mixed state with the increasing channel length. To facilitate an easier comparison of the main trends of the mixing performance between the various mixing structures, we fitted the experimental MI(x), x from 0 to 1 cm, with a two-exponential equation to describe the equilibration process. Thus, modeling the MI as a double exponential increase to saturation using four parameters follows Equation (2):

MI *=* a(1 − e^−bx^) + c(1 − e^−dx^), (2)

The parameters a, b, c and d were obtained by fitting MI(x), Equation (2), to the experimentally observed data using the constraints, a, b, c and d greater than 0 and either a + c < 1 or a + c = 1 (SigmaPlot). The analysis shows that a double exponential function with 4 parameters, where a + c < 1, was adequate to account for the trends in MI(x) while at the same time limiting the number of parameters. Further aspects of the selection of this procedure was reported previously [56]. The image processing, calculation of the MI based on the observed fluorescence distributions and the further representation of MI using Equation (2), were conducted for the layers adjacent to a possible sensor location (termed sensor layer, SL, adjacent to the bottom in Figure 1c) or were based on the mean over the height of the channel (channel layers, CL). Additionally, we refer to all layers, i.e., the channel layers with the extension of the grooves, as all layers (AL).

### 2.5. Statistics

Differences between micromixer design groups with respect to robustness in mixing performance when varying parameter dimensions were tested statistically based on the variance of MI between the groups. Thus, an F-test was used, rejecting null hypotheses when the ratio of the variances, F, were larger than F_α/2, N1-1, N2-1_, where α is the significance level and N1-1 and N2-1 are the degrees of freedom.

## 3. Results and Discussion

**Figure 2:**
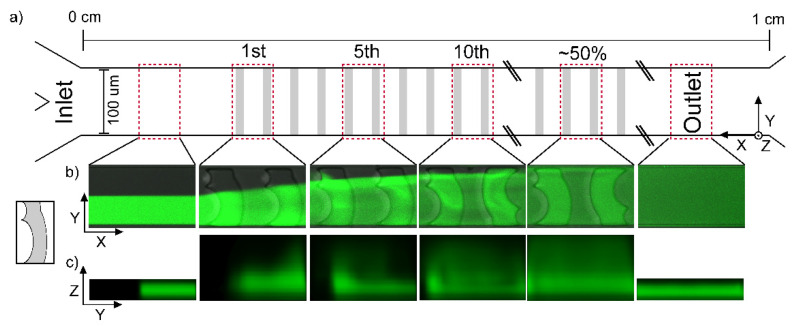
(**a**) Schematic illustration of a mixing channel with arbitrary mixers (grey rectangles) for identification of location of mixing structures from the inlet (left) to the outlet (right) with the location of image acquisition regions (red dashed rectangles). (**b**) Confocal projection of fluorescence intensity along the W_2_ DCMS 1 channel within the selected regions (XY view with intensity averaged over all Z layers) and (**c**) projection of fluorophore perpendicular to the channel direction (sum of intensities along X in each region leading to a YZ-projection). The fluorescence intensity projections were deduced by the custom-designed image processing routine of the confocal XYZT-stacks acquired.

**Figure 3:**
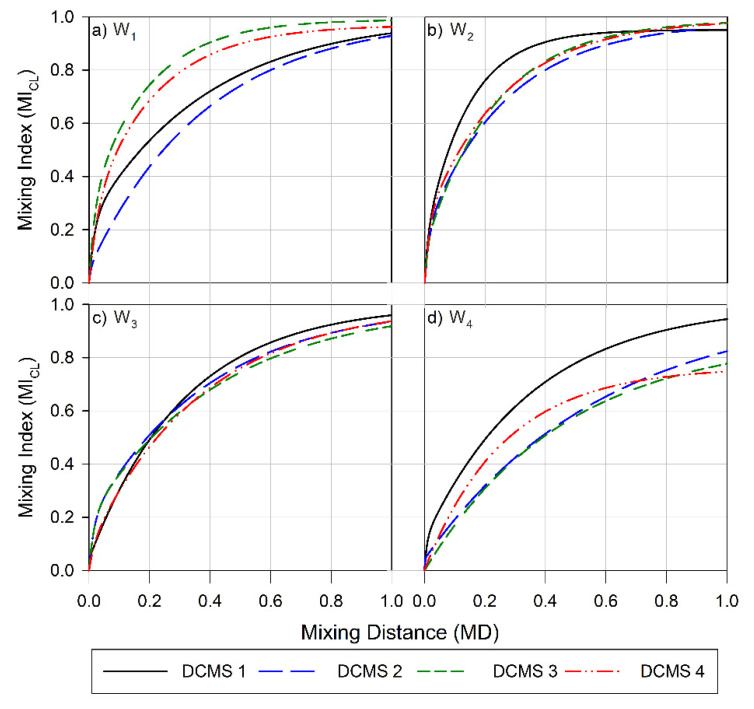
Mixing index for the channel layers (CL) versus the channel length for (**a**) Wafer 1, (**b**) Wafer 2, (**c**) Wafer 3, and (**d**) Wafer 4. Data obtained for channels with parameter design variables (PDV, Table 1) are depicted as follow: PDV 1 is a black solid line, PDV 2 is a blue long-dashed line, PDV 3 green is three short-dashed lines and PDV 4 is a red dashed-dot-dot line.

To validate the mixing and surface coverage efficiencies of the designs, a qualitative analysis of the flow pattern (Figure 2) and a quantitative analysis for the MI (Figure 3) were employed. The development of the flow patterns was realized in both the transverse and the axial direction, resulting in the possibility of a pseudo 3D analysis. The addition of the double curve design changes the flow pattern drastically, compared to the CMS previously studied [56], in both dimensions (Figure 2).

In the following, the flow pattern in the DCMS describes the case where the Y-channel inlet initially exposes the fluid flow with the fluorescein solution to the long curve of the DCMS (Figure 2b). The DCMS mixing structure induces change in the observed flow pattern for all Z-layers as follows. The DCMS, W_2_ DCMS 1, induces a distance dependent enhancement of the fluorescence on the side initially loaded with water and no enhanced fluorophore within the grooves on that side. This gradual spreading, as observed from the pattern in the XY planes, is further exemplified after 5 and 10 structures where the fluorescently depleted regions remain on the top for the DCMS (Figure 2b). Halfway downstream of the channel (e.g., 50%, Figure 2), there is still an observable region of lower intensity in the projections along the channels, which no longer exists at the outlet, where homogeneous distributions are observed (Figure 2b).

**Figure A1:**
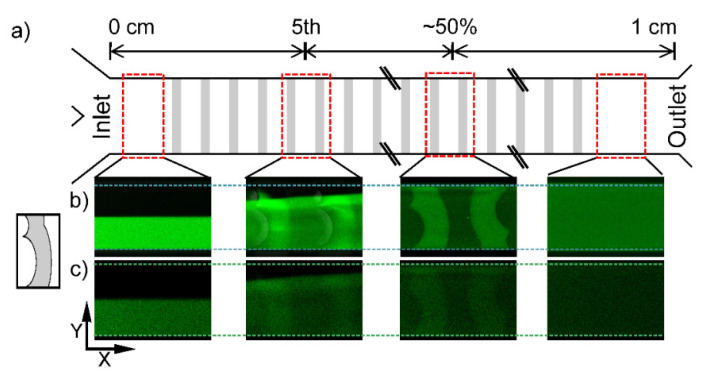
Schematic illustration of a mixing channel with arbitrary mixers (black dashed rectangles) for location purposes from the inlet (left) to the outlet (right). (**a**) Schematic illustration of a microfluidic mixing channel containing DCMS.Confocal XY view averaged intensity for (**b**) all Z levels and (**c**) the sensor layer. XYZT-stacks were acquired after each structure until the 10th structure, subsequent stacks were recorded after each structure. For mixing channels with fewer than 50 structures, stacks were recorded after every fifth structure.

The developing flow pattern along the channels can also be observed in the YZ projections (Figure 2c), where the DCMS structures induces a combined migration (1st mixing structure) and a region of increased fluorescence in the part of the channel opposite the injection side (5th mixing structure). The latter could indicate that a swirling motion has taken place. Such a rotational flow is more evident after 10 structures, although a more heterogeneous distribution remains after 50% channel length. A similar analysis was performed for the surface layer (SL) (Figure A1) of the DCMS structures as a basis for comparison to the bulk flow. The developing homogenization process, as viewed in the XY projections, are considered to be similar for the SL as compared to the bulk movement. While the above development of the flow pattern is observed when the Y-channel inlet guiding the fluorescently labelled stream to the initial long curve is observed, reverting the labelled fluid to the alternative inlet will generate the “mirror” image of the observation reported in Figure 2b. Thus, the unlabeled part of the developing pattern reflects the initial distance-dependent reduction of the spread across the channel (Figure 2b 1st) for the DCMS structures.

Some differences in the developing flow pattern of DCMS as compared to that of the previously reported single-curved mixing structures (CMS) [56] include an earlier indication of swirling flow already within the 1st mixing structure of CMS as compared to the 5th mixing structure for the DCMS. The initial distance-dependent depletion of fluorescence intensity from the side of the channel where the fluorophore was injected in the case of CMS was compensated by the swirling motion, yielding an overall more efficient homogenization as a function of distance than the DCMS.

**Figure A2:**
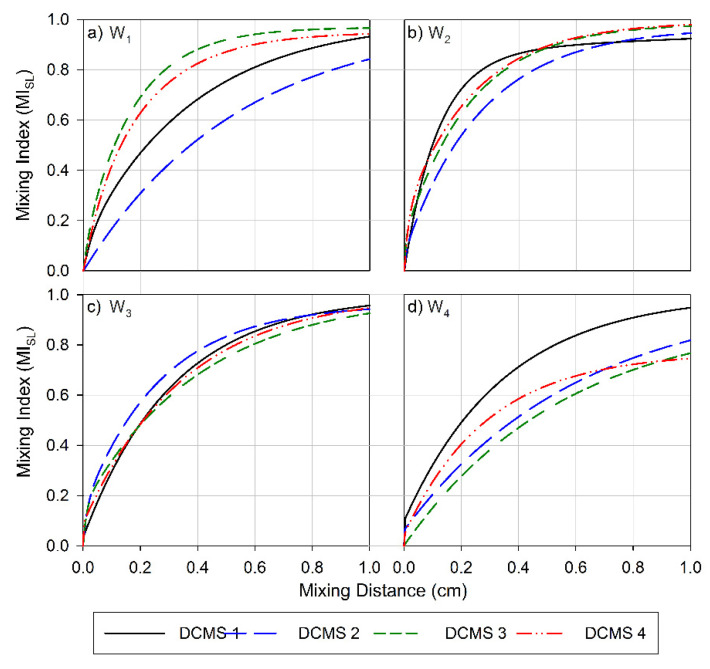
Mixing index at the surface layer versus the channel length for (**a**) Wafer 1, (**b**) Wafer 2, (**c**) Wafer 3 and (**d**) Wafer 4. Data obtained for channels with parameter design variables (PDV, Table 1) are depicted as follow: PDV 1 is a black solid line, PDV 2 is a blue long-dashed line, PDV 3 is a green three short-dashed line and PDV 4 is a red dashed-dot-dot line.

The flow visualization provides a qualitative approach to compare efficiencies between different structures. Quantitative determination of the MI was also performed as a further expansion to be used for comparing mixing performance. Employing Equations (1) and (2), we gathered the MI for the various DCMS micromixers with various parameter values (Table 1). These are presented in terms of which H_C_:H_PM_ ratio they belong to (W_1_, W_2_, W_3_ or W_4_) and whether we have used the average bulk flow, represented as the channel layers (CL), or the fluid layer adjacent to the channel surface, represented as the surface layer (SL), as a basis for the determination of MI (Figure 3 and Figure A2).

The efficiency of the designs to enhance mixing (Figure 3) and to facilitate surface coverage (Figure A2) was found to a depend on the height ratios of the channel. Increasing this ratio from 1:1 (W_1_, Figure 3a) to 1:2 (W_2_, Figure 3b) increased the overall average efficiencies realized. The MI observed at the outlet, 1 cm downstream from the inlet, was 0.96 (range 0.06) for W_1_ (Figure 3a), 0.92 (range 0.03) for W_2_ (Figure 3b),0.94 (range 0.04) for W_3_ (Figure 3c) and 0.82 (range 0.20) for W_4_ (Figure 3d). A similar trend in the average efficiencies was also observed for the surface layers (SL) (Figure A2), where the MI realized for W_4_ showed the lowest average MI at the outlet and the largest range. However, this dependence was not as prominent as for the previously reported CMS [56].

The pitch of the structures (S + D, Table 1) was also found to strongly influence the length dependence of the MI. The more strongly distance-dependent increase in mixing and MI at the outlet (1 cm from the inlet) observed for DCMS 3 on W_1_ (Figure 3a) as compared to DCMS 1 and 2 on the same wafer correlates with the changes in pitch between these structures. This low MI and high MD observed in this case for DCMS 1 and *2* could be due to the structures having a too-narrow pitch (S + D), resulting in inefficient flow distributions. As the structures becomes wider and further apart, there is a large increase in MI and a decrease in MD, and this trend was also observed for the CMS design [56]. These efficient designs were comparable to those realized in W_2_ and W_3_ in achieving efficient mixing.

However, a further increase in the pitch, as observed on W_4_ (Table 1, Figure 3d) resulted in a large decrease in MI and an increase in the range of observed efficiencies. In this instance, only W_4_ DCMS 1 resulted in an MI comparable to the most efficient design (W_1_ DCMS 3). This decrease in efficiency, as the structures become wider and deeper, could be due to the formation of dead volumes within the structures, thereby lowering their ability to perform as efficient mixers. However, this is not in line with that reported for the CMS group, where the least efficient channels were those realized on wafers with geometry as W_1_, and here especially, CMS 1 and CMS 2 [56].

The channels showed a similar MI at the outlet for all DCMS 1 designs (mean 0.95, range 0.02), where the ratio between H_PM_: D: S was 1:1:1. A similar trend in MI was also observed for all designs where the pitch was 120 µm (Table 1) (mean 0.95, range 0.03), whereas an increase in pitch to 240 µm resulted in an average MI of 0.90 (range 0.20). This indicates that there is not a particular design parameter that can be universally applied to achieve the best mixing performance. From the designs evaluated, the three main parameters to consider are the height (H_PM_), depth (D), and spacing (S) of the DCMS.

A similar relationship between H_PM_:D:S was also observed for the surface layer (SL) (Figure A2), thus indicating an apparent similar developing fluorescence distribution at the surface and throughout the channel. However, a notable difference was observed for DCMS 2 from W_1_ and W_3_. In the case of W_1_ DCMS 2, the rate of homogenization of the fluid layer at the surface (Figure A2a) was lower than for the rest of the bulk fluid (Figure 3a), thereby resulting in lower MI values at the same distance from the inlet (i.e., MD_0.6_ = 0.49 cm (SL) and 0.33 cm (CL), whilst at 1 cm, the MI = 0.84 (SL) and 0.93 (CL)). The SL for W_3_ DCMS 2, on the other hand, showed a slightly faster initial rate of fluid homogenization and a larger MI at the outlet compared to the bulk flow in CL. In this instance, the homogenization throughout the channel required an extra 0.06 cm to reach an MI of 0.6 compared to the SL, although the MI at 1 cm was 0.94 for both.

These variations in MI and mixing rate (as determined by the variations in the mixing distance required to reach an MI of 0.6) show the difficulty in predicting flow behavior depending on changes to different geometrical parameters, as well as the relationship between the fluid environment at the surface of the channel compared to an average of the bulk flow throughout the channel volume.

**Figure 4:**
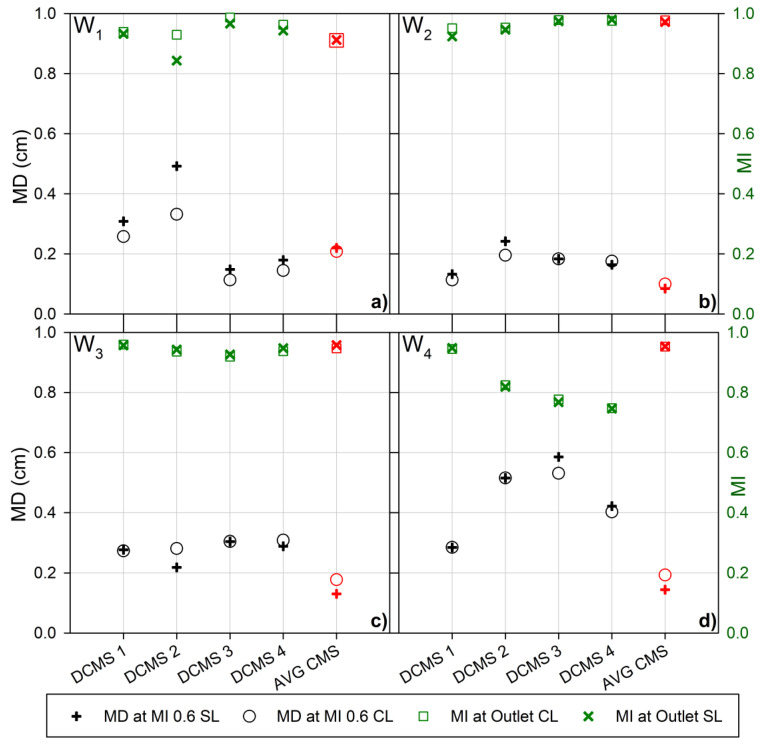
Mixing distance to achieve an MI of 0.6 (60%) (left Y-axis, black) for the channel layers (black ○) and the sensor layer (black **+**) and the mixing index at the outlet (right Y-axis, green) for the channel layers (green □) and the sensor layer (green **×**) for the microfluidic channels with passive mixing structures, as indicated. The parameter values for the microfluidic channels with mixing structures with parameter design variables (Table 1) as prepared from (**a**) Wafer 1 (W_1_), (**b**) Wafer 2 (W_2_), (**c**) Wafer 3 (W_3_) and (**d**) Wafer 4 (W_4_), respectively. The average values for the previously reported curved mixing structure (AVG CMS) [56] is represented in red.

The data obtained showed a strong correlation between the mixing distance (MD) required to achieve an MI of 0.6 (MD_0.6_), and the MI reached at the outlet, where a smaller MD_0.6_ typically leads to a larger MI at the outlet. Since a short MD_0.6_ and a large MI at the outlet both indicate efficient mixing, such a correlation can be expected. However, this was not always the case, as seen for W_4_ DCMS 2 and W4 DCMS 4 (Figure 4d). In this instance, W_4_ DCMS 2 and W_4_ DCMS 4 resulted in an outlet MI of 0.82 and 0.75, respectively, which was unexpected as the corresponding MD_0.6_ values were 0.52 cm and 0.40 cm. Therefore, although W_4_ DCMS 4 possessed a more rapid initial mixing rate, the resulting MI at the outlet was lower than for other structures with similar initial rapid mixing.

A similar trend was also observed for W_2_ DCMS 1, where a rapid initial increase in MI resulting in an MI of 0.9 after roughly 0.3 cm, produced the lowest MI at the outlet, for the same wafer designs (Figure 3b). This indicates the presence of different length dependencies of the processes, inducing the homogenization throughout the channel or different optimal sensor locations. For example, comparing the MI for CL and SL illustrates this, where W_1_ DCMS 2 (Figure 4a) clearly indicate a difference in the MI and MD_0.6_ analyzed based on CL or SL. Nevertheless, the trend in performance still holds true, as the CL shows a shorter MD_0.6_ and a larger MI compared to the SL.

The average values for the previously reported CMS design provide an easy comparison between the two design groups, as the DCMS is a direct evolution of the CMS design. The passive mixing channels fabricated based on W_2_ and W_3_ (Table 1, Figure 4b,c) resulted in the lowest range of efficiencies, largely due to the low performance seen for the DCMS group on W_4_ (Figure 4d). The low performance for the DCMS group was surprising due to the otherwise low spread in the high efficiencies for the CMS and HBS previously reported [56].

**Figure 5:**
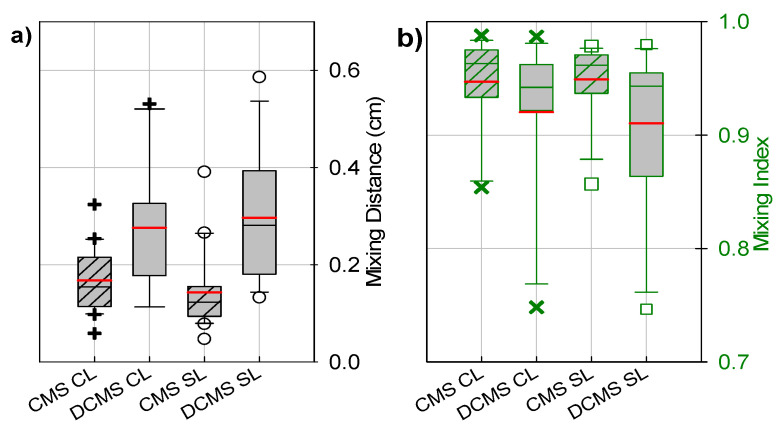
Boxplots depicting (**a**) a range of mixing distances required to achieve a mixing index of 0.6 (black) and (**b**) a range of mixing indices at the outlet (green) for the channel layers (CL) (black **+** and green **×**) and surface layer (SL) (black ○ and green □) for the CMS (striped box) and DCMS (gray box) groups. The red and black/green lines within the boxes depict the mean and median values, respectively. The box corresponds to the interquartile range (50% of the data), whilst the lines (┬ ┴) represent 1.5× the interquartile range. The CMS data were obtained from our previous study [56].

Overall, the passive mixing channels with the CMS group yielded an MI after 1 cm (outlet) and MD_0.6_ (Figure 5a,b) that was less sensitive to altered parameter values than the DCMS group. The CMS group resulted in the largest MI and the lowest MD_0.6_, both for the CL and the SL. On average, the addition of the second curve resulted in lower MIs and larger MD_0.6_ (Figure 5a,b), as analyzed both throughout the channel and for a layer adjacent to the possible sensor location, CL and SL, respectively. This was mainly due to the decrease in mixing performance observed on W_4_ (Figure 4d), and the increase in MD_0.6_ from W_1_ and W_4_ (Figure 4a,d). The CMS group was found to yield a statistically significant different variance (smaller) of the MI at the outlet than the DCMS (at level of significance α = 0.05).

The mixing efficiencies reported here for the asymmetric double curved mixing structures are also within a range obtained for other passive microfluidic mixers utilizing similar mixing lengths and Re numbers. Hossain et al. optimized the SHM placed on the top and bottom wall of a microchannel and achieved a simulated MI range of 0.68 to 0.93 for various design variables [42]. Kim et al. designed a barrier-embedded micromixer that achieved an MI of 0.40 at Re = 120 for a mixing length of 21 mm, although this was reduced to 0.23 for Re = 1 [57]. Comparing the present results also to include 3D dimensional mixers, the maximum MI reached was often over 0.9 [10], such as the serpentine crossing channel resulting in an MI ranging from 0.9–0.99 over an Re range of 0.01–120 [58,59,60]. Nevertheless, the 3D mixing designs such as the serpentine crossing channel do not readily support combination with biosensors integrated in the designs. Thus, the overall aim of those designs is directed to homogenization of the liquid at the outlet and does not support the feasibility for sensor integration, compared to the designs realized in the present study.

## 4. Conclusions

The results on the mixing index (MI) and the initial rate of mixing for a newly fabricated passive ceiling mixing structure group show a large dependence on the geometry of the structure. The addition of a second curved structure to the previously studied curved mixing structure (CMS) group [56] resulting in the novel double curved mixing structure (DCMS) had a drastic effect on the flow pattern induced by the mixers and the resulting efficiencies. The MI for the mixers were determined using confocal microscopy, where the homogenization process between water and fluorescein solution were analyzed for the specific regions of the channel volume, as well as for the sensor layer. This provided a description for the flow and progression throughout the channel in the XY and YZ planes of the channel.

The initial mixing rate was determined from the required mixing distance (MD) necessary for a design to achieve an MI = 0.6 (MD_0.6_), where MI = 1 would be complete homogenization. Varying the geometric parameter channel height (H_C_), mixing structure depth (D), spacing (S) and height (H_PM_) resulted in a range of mixing efficiencies, where the three most important parameters to consider for the DCMS design were H_PM_, D and S.

The most efficient DCMS, W_1_ DCMS 3 resulted in an MI of 0.99, whilst the least efficient, W_4_ DCMS 4, was 0.75, leaving a range from 0.75 to 0.99 for the MI at the outlet (after 1 cm of mixing). We compared these results to the CMS group reported previously [56], which showed an MI range from 0.99 (W_2_ CMS 1) to 0.85 (W_1_ CMS 2). The more extended overall range of MI at the outlet of the DCMS (0.75–0.99) as compared to that reported previously for the CMS (0.85–0.99) using similar spacings and pitches, indicating that the MI at the outlet are more sensitive to parameter values in the DCMS design than the CMS design. A similar relationship was also experienced at the surface of the channels, where the MD_0.6_ range was 0.11–0.53 cm and 0.06–0.32 cm for DCMS and CMS respectively (Table A1).

Decreasing the height of the channel and the passive mixing structures resulted in an increase in the MI after 1 cm, as well as a reduction in MD_0.6_ for the DCMS group. This could be due to the reduction in potential dead volumes experienced at the larger depth and height of the structure, or an increased efficiency at a larger velocity, as at the same flow rate the velocity of the fluid increases for smaller channels. The higher performance of the CMS could be due to the structure being more capable of inducing chaotic advection compared to the DCMS and the HBS. The DCMS group is therefore more sensitive to variation in its geometric parameters compared to the CMS group.

Here we have provided a large parametric study for the design of double curved passive mixing structure, where an efficient design can be achieved over a variation of channel heights. This provides a guide to design microchannels with efficient mixing.

## Figures and Tables

**Table 1:** Parameter values of microfluidic channels with DCMS in the ceiling of the microfluidic channel. The table lists all combinations of the parameters implemented by the lithography process. The design parameters are schematically illustrated in Figure 1. The master molds on the four wafers were realized for fabrication of channels with total heights: Wafer 1 (W_1_) = 40 µm, Wafer 2 (W_2_) = 60 µm, Wafer 3 (W_3_) = 80 µm and Wafer 4 (W_4_) = 100 µm. Sixteen different channels with design parameter variables were fabricated. All channels had a width (along the y-direction) of 100 µm, and a mixing channel length (x-direction) of 1 cm.

Parameters	Wafer 1 (W_1_)			Wafer 2 (W_2_)			Wafer 3 (W_3_)			Wafer 4 (W_4_)		
Channel height (H_C_)	20			20			40			40		
Passive mixer (PM) height (H_PM_)	20			40			40			60		
Parameter Design Varaible (PDV)	PM Depth (D)	PM Spacing (S)	PM Pitch (D + S)	PM Depth (D)	PM Spacing (S)	PM Pitch (D + S)	PM Depth (D)	PM Spacing (S)	PM Pitch (D + S)	PM Depth (D)	PM Spacing (S)	PM Pitch (D + S)
1	20	20	40	40	40	80	40	40	80	60	60	120
2	20	40	60	40	80	120	40	80	120	60	120	180
3	40	40	80	80	80	160	80	80	160	120	120	240
4	40	80	120	80	160	240	80	160	240	120	240	360

## Data Availability

Relevant data are included in the manuscript and its Appendix A.

## Appendix A

**Table A1:** Range in MI found at the outlet (1 cm) and the mixing distance (MD) required to obtain a mixing index of 0.6. The max and min values (and which design these represent) are presented for the channel layers (CL) and the surface layer (SL), as well as the range. Represented are the two design families compared in this article, CMS and DCMS, as well as a previously studied herringbone-type structure (HBS) presented in our previous article [56].

MI at Outlet	Max_CL_		Min_CL_		Range_CL_	Max_SL_		Min_SL_		Range_SL_
CMS	0.99	W_2_ CMS 1	0.85	W_1_ CMS 2	0.14	0.98	W_2_ CMS 1	0.86	W_1_ CMS 1	0.12
HBS	0.98	W_2_ HBS 3	0.74	W_1_ HBS 2	0.24	0.98	W_2_ HBS 3	0.72	W_1_ HBS 2	0.26
DCMS	0.99	W_1_ DCMS 3	0.75	W_4_ DCMS 4	0.24	*0.98*	W_2_ DCMS 4	0.75	W_4_ DCMS 4	0.23
MD (cm) at MI_0.6_										
CMS	0.32	W_1_ CMS 1	0.06	W_2_ CMS 2	0.26	0.39	W_1_ CMS 1	0.05	W_2_ CMS 2	0.34
HBS	0.66	W_3_ HBS 2	0.11	W_2_ HBS 3	0.55	0.66	W_3_ HBS 2	0.06	W_4_ HBS 3	0.60
DCMS	0.53	W_4_ DCMS 3	0.11	W_2_ DCMS 1	0.42	0.59	W_4_ DCMS 3	0.13	W_2_ DCMS 1	0.46
